# Functional constipation induces bladder overactivity associated with upregulations of Htr2 and Trpv2 pathways

**DOI:** 10.1038/s41598-020-80794-0

**Published:** 2021-01-13

**Authors:** Nao Iguchi, Alonso Carrasco, Alison X. Xie, Ricardo H. Pineda, Anna P. Malykhina, Duncan T. Wilcox

**Affiliations:** 1 Division of Urology, Department of Surgery, University of Colorado Denver School of Medicine, 12700 E 19th Avenue, Aurora, CO 80045 USA; 2 Children’s Hospital Colorado, 13123 E 16th Avenue, B463, Aurora, CO 80045 USA; 3 Children’s Mercy Kansas City, 2401 Gillham Rd, Kansas City, MO 64108 USA

**Keywords:** Molecular biology, Diseases, Pathogenesis, Urology

## Abstract

Bladder and bowel dysfunction (BBD) is a common yet underdiagnosed paediatric entity that describes lower urinary tract symptoms (LUTS) accompanied by abnormal bowel patterns manifested as constipation and/or encopresis. LUTS usually manifest as urgency, urinary frequency, incontinence, and urinary tract infections (UTI). Despite increasing recognition of BBD as a risk factor for long-term urinary tract problems including recurrent UTI, vesicoureteral reflux, and renal scarring, the mechanisms underlying BBD have been unclear, and treatment remains empirical. We investigated how constipation affects the lower urinary tract function using a juvenile murine model of functional constipation. Following four days of functional constipation, animals developed LUTS including urinary frequency and detrusor overactivity evaluated by awake cystometry. Physiological examination of detrusor function in vitro using isolated bladder strips, demonstrated a significant increase in spontaneous contractions without affecting contractile force in response to electrical field stimulation, carbachol, and KCl. A significant upregulation of serotonin receptors, Htr2a and Htr2c, was observed in the bladders from mice with constipation, paralleled with augmented spontaneous contractions after pre-incubation of the bladder strips with 0.5 µM of serotonin. These results suggest that constipation induced detrusor overactivity and increased excitatory serotonin receptor activation in the urinary bladder, which contributes to the development of BBD.

## Introduction

Functional urologic and bowel problems are common in childhood, account for up to 40% of consultations in paediatric urology, and can cause both physical and psychosocial distress for children and families^1^. The clinical spectrum of paediatric lower urinary tract dysfunction (LUTD) is wide, including over- or under-active bladder, frequent- or infrequent voiding, voiding postponement, urgency, enuresis and nocturia. LUTD may be associated with other conditions including neuropsychiatric problems and gastrointestinal dysfunctions, constipation being the commonest comorbidity of LUTD. Constipation is typically defined as infrequent bowel evacuations (less than three bowel movements per week), abnormally large stools, and difficult or painful defecation. Functional constipation is a common problem in childhood with the prevalence of up to one-third of children^2^. Constipated children were reported to be 6.8 times more likely to have LUTD compared to those with normal bowel function, and up to 84% of functionally constipated children suffer from encopresis which is also often associated with LUTD^3–5^. With increasing recognition of the prevailing comorbidity of LUTD and bowel disturbances in children, the condition has been labelled with a standardized term, bladder and bowel dysfunction (BBD) by the International Children's Continence Society in 2014^6^. Due to a diverse spectrum, BBD is often not recognized by child, family or the referring professional, instead, the secondary symptoms of urinary and/or faecal incontinence or urinary tract infection (UTI), prompt the child to be evaluated by specialists^7–9^. Hence the diagnosis of BBD is often delayed, and the treatment mainly being symptomatic management, which increase a risk of prolonged and more serious conditions such as recurrent UTI, faecal impaction, rectal prolapse, vesicoureteral reflux, renal failure, and irritable bowel syndrome^8,10–13^. In addition, clinical studies found that adults with LUTD and/or bowel symptoms often experienced BBD symptoms in childhood, underscoring the importance of recognizing BBD early on and to address both, LUT and gastrointestinal dysfunctions, simultaneously^11,14–16^.

The aetiology of BBD seems to be multifactorial, and the mechanical compression of large stool in the colon or rectum during constipation is considered to be a principal cause for LUTD development^17^. Physical pressure onto the LUT decreases the functional capacity of the urinary bladder, constricts the urethra, thereby resulting in urgency, dysfunctional voiding, urinary retention and urinary incontinence^18^. This explains the improvement of lower urinary tract symptoms (LUTS) following the medical relief of constipation in the majority of cases with BBD symptoms^8,19^. However, other studies found no improvement or persistent LUTS with release of intra-rectal pressure in children with BBD^20–22^.

Normal function of the urinary bladder and gastrointestinal tract depends on their components including the nerves, muscle, connective tissues and mucosa, and the coordinated interactions among them. Prolonged BBD causes muscle instability in both bladder and colon, suggesting that BBD affects the muscles and nerves that control normal bowel and bladder function. One proposed mechanism is the development of bladder-bowel cross-organ sensitization via overlapping neural pathways at both peripheral and central level^23,24^. Other possibility includes histological and/or molecular changes in the tissues including inflammation, fibrosis, alterations in intercellular connections, signalling molecules, receptors and ion channels^15,25–28^. As such, the pathophysiological mechanisms underlying BBD are complex, yet not understood very well. In the current study, we examined the effects of functional constipation on the LUT early in life using a murine model of constipation at young age.

## Materials and methods

### Animals

Male C57BL/6J mice (4-week-old) were divided into two groups: constipation model, and sham group. Constipation was induced as previously described by Heredia et al*.*^29^. Briefly, a lubricated 2.0 mm diameter polyethylene tubing was inserted into the anus of mice (1 cm) under anaesthesia. After injection of 2% lidocaine into the skin next to anal opening, a purse-string suture (size 7–0, Prolene, Ethicon, Somerville, NJ, USA) was snugly placed in the external sphincter region around the tubing, and then the tubing was removed. To create functional constipation model (N = 42), the suture remained in place for four days. Sham operated animals underwent the same procedure except for the immediate removal of the suture after its placement (control group, N = 36). Mice were maintained in the animal facility (14-h light: 10-h dark cycle) with access to water and chow ad libitum. Rectal sutures and perianal area were checked to ensure the integrity of the model. The colorectum and urinary bladders were harvested for examination at four days after the surgery. Urine samples were collected from the bladders (N = 12 per group) and tested with urinalysis strips (U031-131, ACON laboratories, San Diego, CA, USA). All animal procedures were reviewed and approved by the Institutional Animal Care and Use Committee (IACUC) of the University of Colorado Denver. All experiments were performed in accordance with relevant guidelines and regulations, as well in compliance with the ARRIVE guidelines 2.0 (https://arriveguidelines.org/arrive-guidelines).

### Micturition pattern evaluation

In order to evaluate voiding patterns, void spot assays (VSA) were conducted at four days after the surgery, as previously described (N = 22 per group)^30^. Each mouse had free access to water but no food during the 3 h test period. All VSA experiments were started at 10 am. The urine spots on the filter paper were imaged using ultraviolet light on a transilluminator, and the number and area of the spots were analysed using Adobe Photoshop CS6 (Adobe Systems, San Jose, CA, USA). Faecal pellets were collected and air dried at least 16 h following VSA (N = 8 per group), and the number and the weight of pellets were recorded.

### Urodynamic evaluation of bladder function

For urodynamic evaluation, mice underwent surgical catheter implantation in the bladder as previously described^15^ immediately before undergoing the constipation or sham surgery. Cystometry was performed in unanesthetized unrestrained mice 4 days after the surgery (N = 4 per group) as described previously^15^. The tip of the exteriorized bladder catheter located at the base of the mouse neck was connected to a pressure transducer and an infusion pump of the cystometry station (Catamount Research and Development, St. Albans, VT, USA). Room temperature saline was infused into the bladder at the rate of 10 μl/min. Each animal was observed for minimum four voiding cycles of reproducible micturition patterns. Urodynamic values recorded continuously during testing, and four parameters; maximum intravesical pressure at micturition, functional bladder capacity, voided volume, and the number of non-void contractions (NVC) per voiding cycle were analysed using Cystometry Analysis Software (SOF-552, Catamount Research and Development). The NVC were defined as intravesical pressure rises greater than one-third of average maximal voiding pressure in each animal without triggering micturition.

### Physiological evaluation of detrusor function in vitro

In vitro physiological evaluation of detrusor contractility and the baseline spontaneous activity was conducted using freshly isolated bladder strips from mice in each group as described previously^30^. Spontaneous contractions in each bladder strip were collected for 2 min after initial equilibration for 30 min in Tyrode’s solution. Detrusor contractility was examined in the responses to a series of electrical field stimulation (EFS, 70 V, 2–32 Hz), the acetylcholine receptor agonist carbachol (CCh, 1–100 µM), high KCl (125 mM replaced NaCl in Tyrode’s solution), a mixture of purinoceptor agonists (ADP/ATP; ADP-γ-s, ATP-γ-s and αβ-methylene ATP,10 µM each) (n = 14–15 per group). Contractile responses to EFS were also recorded after 20 min of incubation with the following substances: (1) ADP/ATP to desensitize purinoceptors, and (2) atropine (muscarinic receptor antagonist, 1 µM) (n = 10–12 per group). Peak force of the contractile response was calculated in grams of tension per weight of individual bladder strip. Spontaneous contractions in the bladder strips were collected for 2 min after 30 min of (1) initial equilibration in Tyrode’s solution, (2) incubation with 5-hydroxytryptamine (5-HT) at 0.5 µM, and (3) incubation with a mixture of 5-HT at 0.5 µM and a selective Htr2 receptor antagonist, ketanserin (+)-tartrate^31^ (Sigma-Aldrich, St. Louis, MO, USA) at 200 nM (n = 16 per group). All data analyses were performed using PowerLab Lab-Chart version 8.1.9 (ADInstruments, Colorado Springs, CO, USA). Calcium imaging of denuded detrusor sections from each group of mice (~ 6 mm × 10 mm, n = 3) was performed with 10 µM Cal-520 AM (abcam, Cambridge, MA, USA) in Tyrode’s buffer according to the manufacturer’s protocol. The relative amplitude of Ca^2+^ transients was expressed as F/F_0_, where F represents the total fluorescence in an event of fluorescent increase, and F_0_ represent the basal fluorescence. Images were taken every 0.3 s for 3 min.

### Histological analysis

Paraformaldehyde-fixed paraffin sections (5 µm thickness) of the urinary bladders and the distal colon from each group were stained with Masson’s trichrome staining^32^. Area measurement of the distal colon sections was performed for the 4 tissue layers, mucosa, muscularis mucosa, submucosa, and muscularis externa, as well as the oval shaped non-staining area in the mucosa which was considered as mucus vesicles. All measurements were conducted in a blind fashion to avoid biased interpretation. For immunostaining, sections were subjected to heat-induced antigen retrieval (10 mM Tris, 1 mM EDTA, and 0.05% Tween 20, pH 9.0). All antibodies used in this study are listed in Table 1. Control experiments performed without primary antibodies showed neither nonspecific labelling nor cross-reactivity between secondary antibodies. Sections from at least three animals in each group were analysed for reproducibility.

**Table 1:** Antibodies.

*Primary*	Vendor and Catalog No	Application and dilution	Validation
Desmin	Novus Biologicals, NB120-15200	1:200 (IF)	^26^
Gapdh	Santa Cruz Biotechnology, sc-32233	1:500 (WB)	
	Proteintech, HRP-60004	1:5,000 (WB)	
Htr2a	Santa Cruz Biotechnology, sc-166775	1:100 (IF)	^48^
Htr2c	Santa Cruz Biotechnology, sc-17797	1:100 (IF)	^49^
	LSBio, LS-C386171	1:500 (WB)	Supplement 1
Uchl1	Santa Cruz Biotechnology, sc-23852	1:50 (IF)	^50^

*Secondary*	Conjugate	Vendor and Catalog No	Application and dilution
Goat IgG	DyLight488	Rockland Immunochemicals, 605-741-125	1:5,000 (IF)
Mouse IgG	HRP	Jackson ImmunoResearch, 715-035-150	1:10,000 (WB)
	DyLight549	Rockland Immunochemicals, 610-142-002	1:5,000 (IF)
	DyLight549	Rockland Immunochemicals, 610-742-124	1:5,000 (IF)
Rabbit IgG	HRP	Novus Biologicals, NB7160	1:10,000 (WB)
	DyLight488	Rockland Immunochemicals, 611-141-122	1:5,000 (IF)

### Gene expression analysis

Total RNA isolated from the urinary bladders (N = 5 per group) was transcribed into cDNA and used in real-time quantitative PCR (qRT-PCR) as previously described^15^. Expression levels of each gene were calculated as fold changes based on ∆∆Ct values. Data were normalized to the mean of three housekeeping genes: β-actin (*Actb*), Glyceraldehyde 3-phosphate dehydrogenase (*Gapdh*) and TATA box-binding protein (*Tbp*). Western blot analysis was performed to assess the protein expression level as previously described (N = 4 per group)^15^. The antibodies used in this study are listed in Table 1. The signals specific for each antibody were quantified using Fiji ImageJ software (Version 1.53c, National Institutes of Health, Bethesda, MD, USA) and normalized with Gapdh.

### Measurement of serum serotonin levels

Blood serum samples were collected from each group of mice (N = 15 per group) with BD Microtainer serum separator tubes (Becton, Dickinson and Company, Franklin Lakes, NJ, USA) and flash frozen. Serotonin ELISA Kit (Aviva Systems Biology Corporation, San Diego, CA, USA) was used to quantify serotonin concentration in mice serum samples. All ELISA measurements were made in duplicate.

### Statistical analysis

All data were analysed using two-tailed unpaired t-test using GraphPad Prism 8.4.3 (GraphPad Software, La Jolla, CA, USA) between two groups. GraphPad outlier calculator (GraphPad Software, https://www.graphpad.com/quickcalcs/Grubbs1.cfm) was used to detect outliers, which were excluded from the analysis in detrusor physiology test in vitro. A probability value of p < 0.05 was regarded as significant. Results are expressed as means ± standard error of the mean (SE).

## Results

### Functional constipation increases urinary frequency and bladder instability in mice

**Figure 1:**
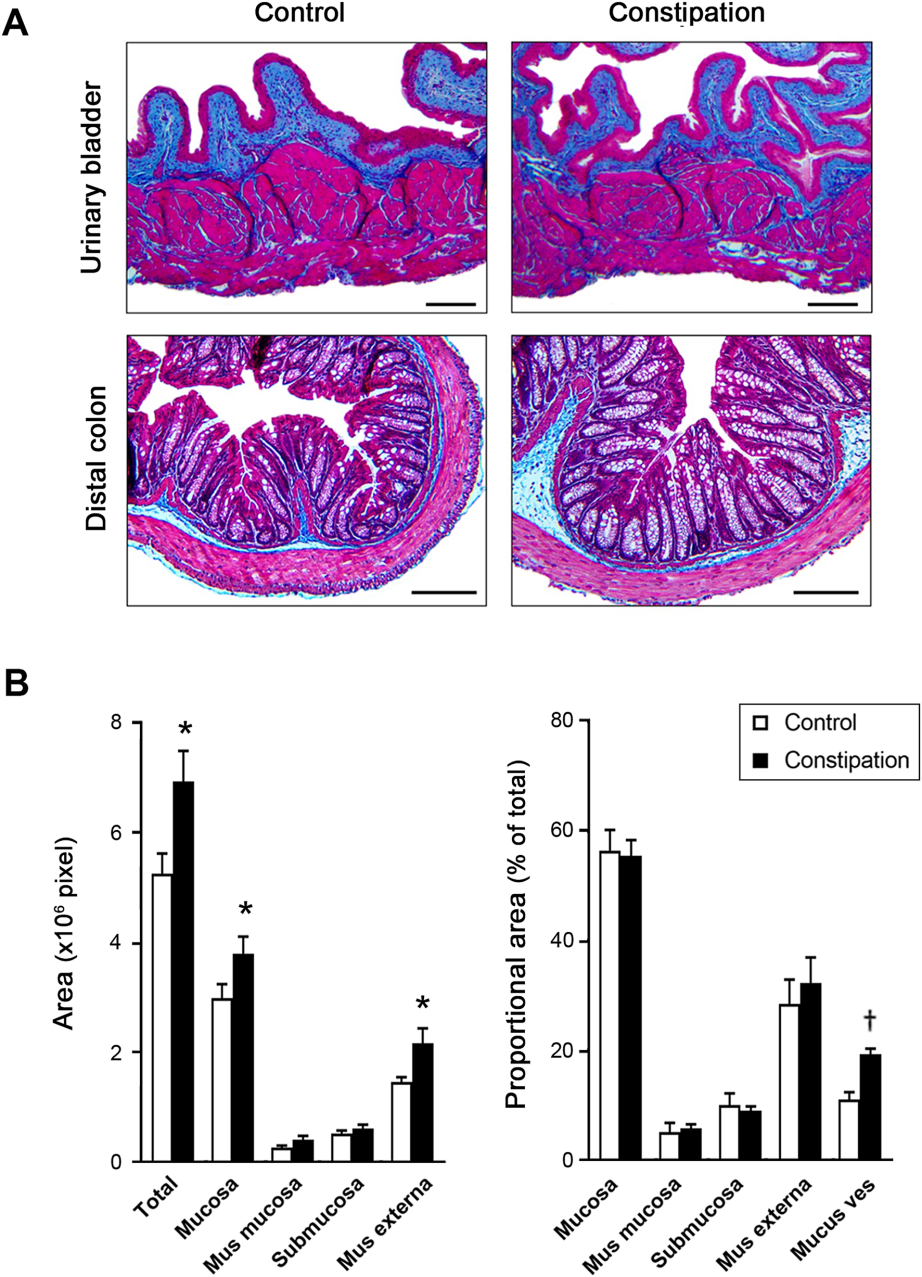
Bladder and colon histology. (**A**) Representative images of cross sections of the bladder (upper panels) and colon (lower panels) from mice in the control (left) and the constipation (right) groups. Masson’s trichrome staining: Collagen is represented by the blue staining. Bars, 200 µm. (**B**) The area measurement of total colon tissue section and the four layers (mucosa, muscularis mucosa, submucosa and muscularis externa) of the colonic wall from each group of mice (left). The proportional area of each layer relative to the entire tissue section and the mucus vesicles relative to the mucosa (right). Mean ± SE. *p < 0.05, ^†^p < 0.0005 vs. the control group. Figures were prepared using Adobe Photoshop CS6 and GraphPad Prism 8.4.3 (https://www.graphpad.com/scientific-software/prism/).

**Figure 2:**
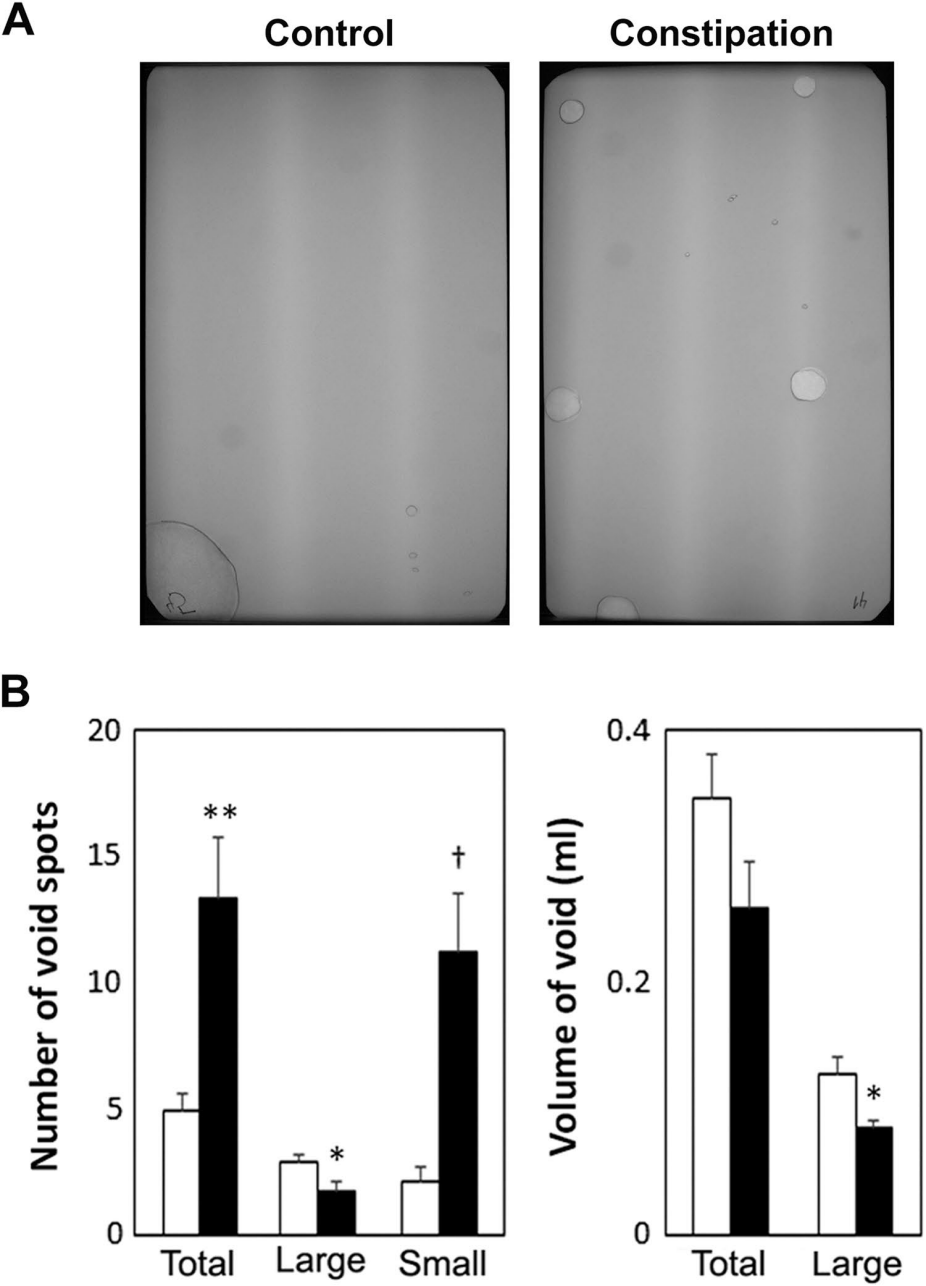
Micturition patterns at 4 days after the surgery. (**A**) Representative void spot assay images from each group. (**B**) Number of the urine spots (left), of large (≥ 50 μl) and small voids (< 50 μl). The voided volume (right) of total and per void in the large void spots. White and black bars represent the control and the constipation mice, respectively. Mean ± SE. *p < 0.05, **p < 0.005, ^†^p < 0.0005 vs. the control mice. Figures were prepared using Adobe Photoshop CS6 and GraphPad Prism 8.4.3 (https://www.graphpad.com/scientific-software/prism/).

**Figure 3:**
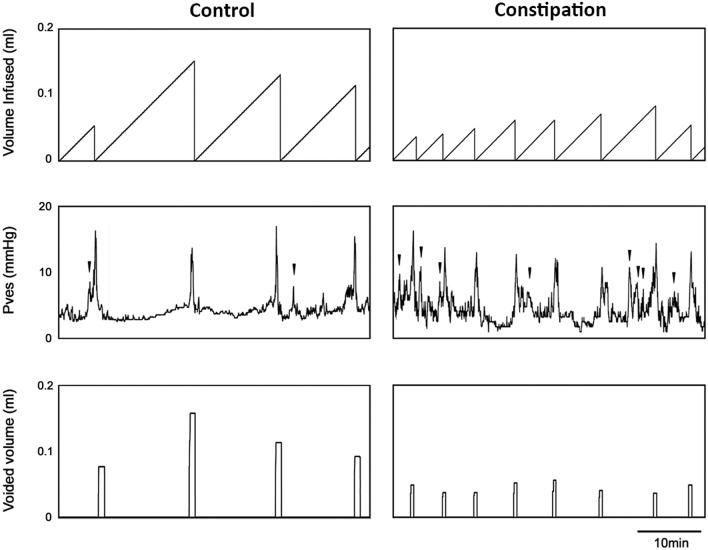
Functional bladder analyses in cystometry. Representative cystometrogram trace from unanesthetized, unrestrained mice in the control (left) and the constipation group (right) during a continuous intravesical infusion (10 μl/min) of room temperature saline. Volume infused (top), intravesical pressure (Pves, middle) and voided volume (bottom) are shown. Arrowheads indicate examples of non-voiding bladder contractions. Figures were prepared using Cystometry Analysis Software (SOF-552, https://www.med-associates.com/product/cystometry-analysis-data-analysis-software/) and Adobe Photoshop CS6.

Partial obstruction of the external anal sphincter caused a growth retardation, and a profound enlargement of the colorectum with faecal impaction in juvenile mice (Table 2). The bladder-body weight ratio and morphology of the bladder were comparable between the groups. No signs of fibrosis, inflammatory cell infiltration or disturbance in the organ walls was detected in both the bladder and the distal colon from both groups of mice (Fig. 1A). However, the total area of the distal colon sections was significantly larger in the constipation group compared to the control. The area analysis of each layers in the colon showed that all 4 layers, mucosa, muscularis mucosa, submucosa, and muscularis externa, were increased in a similar level. The proportion of each layer in the corresponding section was similar between the two groups. We also observed a marked increase in the oval shaped structure in the mucosa which is likely mucus vesicle in the goblet cells in the colon sections from the constipation group when compared to the control group (Fig. 1B). In addition to the faecal impaction, the faecal output during VSA experiments was decreased to less than a half in the experimental group compared to the sham operated control group (4.0 ± 1.0 mg vs. 8.6 ± 0.5 mg, p = 0.0025). Furthermore, the sign of faecal incontinence (loose stool spots) was observed on filter papers in 10 mice from the constipation group, but none from the control group. These results verified that our murine model replicated clinical characteristics of functional constipation in children. Urinalysis showed normal values for all parameters (ascorbic acid, glucose, bilirubin, ketones, specific gravity, blood, pH, protein, urobilinogen, nitrites, leukocyte, albumin, and creatinine), in all urine samples tested (N = 12 per group), indicating that no UTI occurred during the study period. The total number of urine spots in the constipation group was 2.7-fold higher than in the control group (p = 0.0016). Mice with constipation voided more frequently in small volume (< 50 µl per void, 11.2 ± 2.4 vs. 2.1 ± 0.6, p = 0.0005), while less large voids (≥ 50 µl per void, 1.7 ± 0.4 vs. 2.8 ± 0.3, p = 0.024) compared to the control group (Fig. 2). The volumes of total void and the mean of the large urine spots were decreased in the constipation group compared with those in the control animals (259 ± 36 µl vs. 347 ± 33 µl, p > 0.05 and 84 ± 6 µl vs. 126 ± 13 µl, p = 0.012, respectively). To further evaluate how constipation impacts LUT function, urodynamic studies were conducted in each group of animals. Urodynamic parameters were clearly distinguished between the two groups (Fig. 3 and Table 3). Functional bladder capacity was significantly decreased by approximately 30% in the constipation group in comparison to the control (p = 0.041), consistent with the data from VSA experiments. The voiding efficiency and the maximal intravesical pressure at micturition (Pves max) were comparable between the two groups. Intravesical pressure traces in the constipation group revealed bladder instability with frequent NVCs along with continuous small spikes of pressure which were minimal in the control group (p = 0.006).

**Table 2:** Morphological parameters of the urinary bladder and colon in the control and the constipation groups.

	Body (g)	Bladder (% of body)	Colon			Stool (mg)
			Weight (mg)	Length (mm)	Width (mm)	
Control	16.6 ± 0.9	0.09 ± 0.00	167 ± 7	6.5 ± 0.2	1.4 ± 0.1	101 ± 12
Constipation	13.4 ± 0.6**	0.10 ± 0.00	273 ± 22**	6.6 ± 0.2	3.1 ± 0.2**	349 ± 40**

Mean ± SE, **p < 0.005 vs. the control group.

**Table 3:** Comparison of urodynamic parameters between the groups.

	Infused vol. (µl)	Void vol. (µl)	Pves max (mmHg)	Voiding efficiency (%)	Non-void contractions
Control	106 ± 11	109 ± 6	17.0 ± 0.5	105 ± 8	0.1 ± 0.1
Constipation	79 ± 5*	72 ± 7^†^	15.6 ± 2.0	99 ± 7	0.8 ± 0.2*

The constipation group showed a significant decrease in bladder capacity, voided volume, and a significant increase in the number of non-void contractions compared to the control group (N = 4 per each group). Mean ± SE, *p < 0.05 and ^†^p < 0.005 vs. the control group, Pves max, maximum intravesical pressure at micturition.

### Functional constipation induced detrusor overactivity

**Figure 4:**
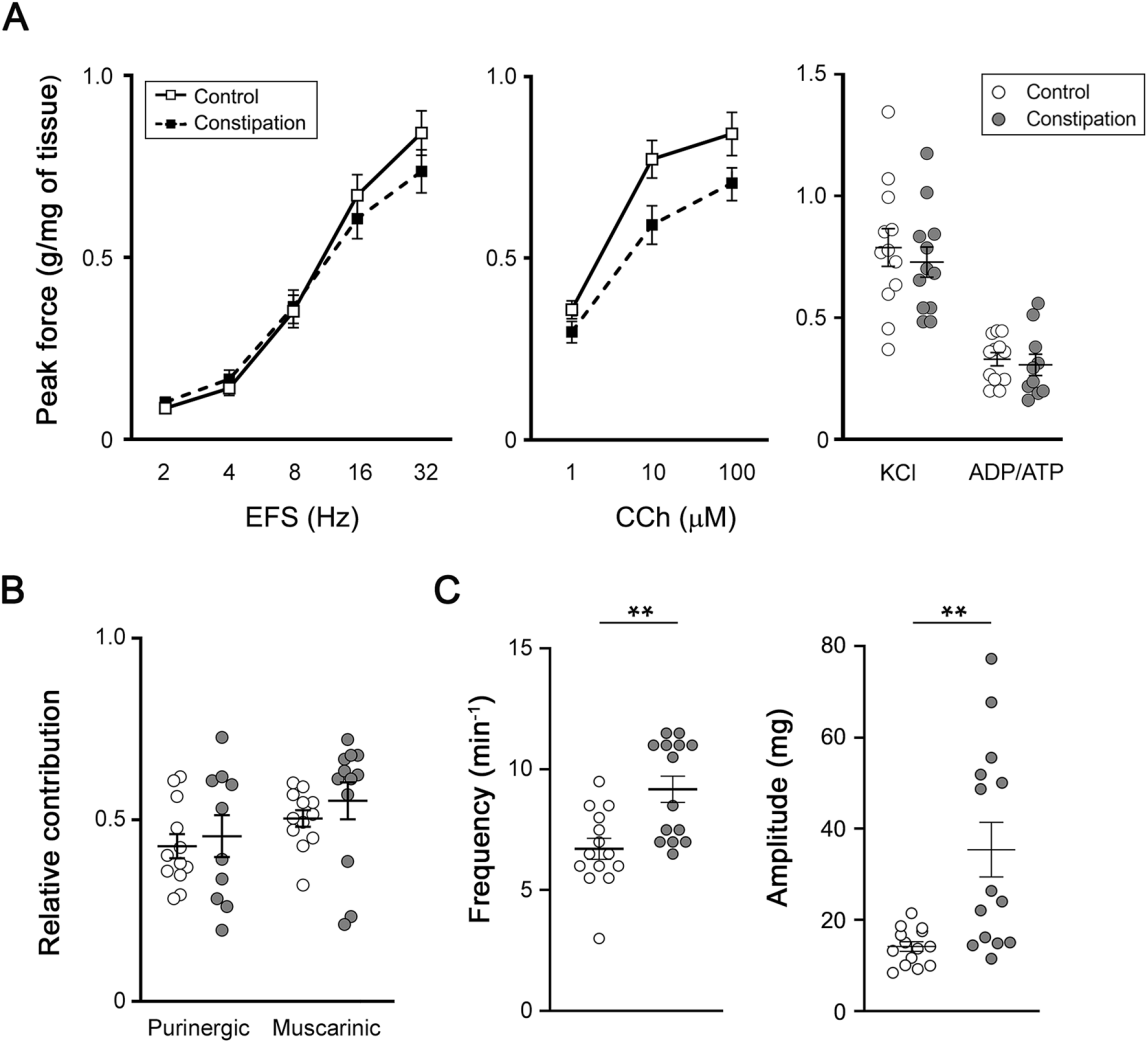
Detrusor contractility evaluation. (**A**) Peak contractile force in response to electric field stimulation (EFS, left), carbachol (CCh, centre), and KCl and ADP/ATP (right). (**B**) Relative contribution of purinergic and muscarinic pathways to EFS-evoked contractility. The force was normalized with tissue weight. (**C**) The frequency (left) and amplitude (right) of spontaneous contractions. White and grey circles represent the control and the constipation groups, respectively. Mean ± SE. **p < 0.005, vs. the control mice. Figures were prepared using Adobe Photoshop CS6 and GraphPad Prism 8.4.3 (https://www.graphpad.com/scientific-software/prism/).

Bladder strips showed comparable contractile responses to EFS, CCh, ADP/ATP and KCl between the two groups. Inhibition of purinergic or muscarinic receptors suppressed EFS-evoked contractile response at similar level in bladder strips from both groups (Fig. 4A,B). However, the analyses of basal activity of the bladder strips revealed a profound increase in spontaneous contractions in the constipation group. Both frequency and amplitude of the spontaneous contractions were elevated compared to the control group (9.2 ± 0.5 vs. 6.7 ± 0.4 min^−1^, p = 0.0015, and 35.6 ± 5.8 vs.14.2 ± 1.0 mg, p = 0.0013, respectively) (Fig. 4C). Likewise, a significant elevation of unprovoked spontaneous intracellular Ca^2+^ transients was observed in detrusor myocytes from the constipation group compared to those from the control animals (incidence, 6.7 ± 1.7 min^−1^ vs. 3.4 ± 1.2 min^−1^, p = 0.037, and F/F0, 7.9 ± 1.6 vs. 3.8 ± 0.8, p = 0.044).

### Functional constipation induced an upregulation of serotonin receptors in the bladder

**Figure 5:**
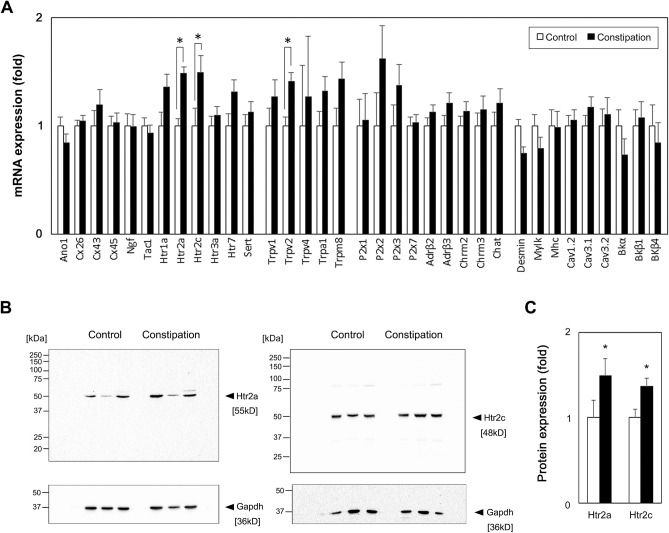
Gene expression analysis in the bladders. (**A**) The level of mRNA expression of each gene is presented as the fold difference to that in the control group (N = 5 per group). (**B**) The protein expression comparison. Representative Western blotting results for Htr2a (top left), Htr2c (top right) and Gapdh (bottom). (**C**) Summary of relative protein expression levels of Htr2a and Htr2c normalized with Gapdh (right). Mean ± SE, *p < 0.05 vs. the control group. Figures were prepared using Adobe Photoshop CS6 and GraphPad Prism 8.4.3 (https://www.graphpad.com/scientific-software/prism/).

**Figure 6:**
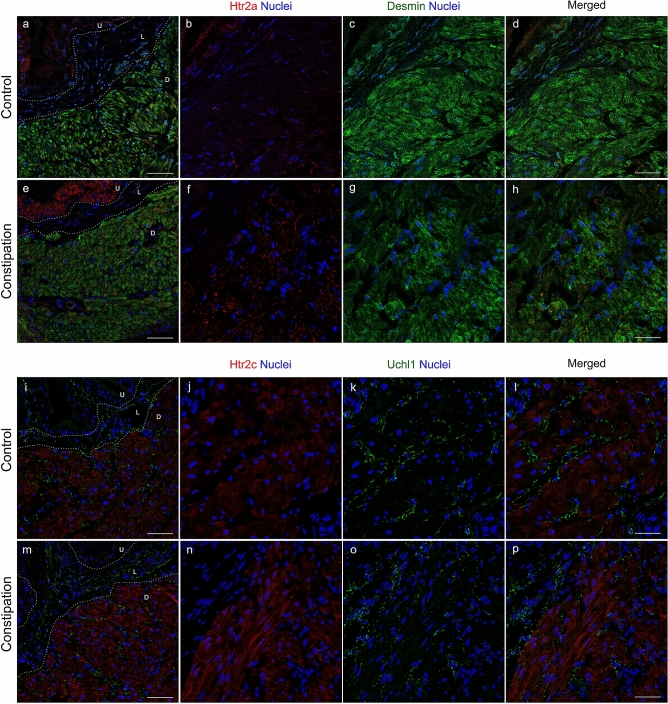
Distribution of Htr2a and Htr2c in the bladders. Representative immunohistochemical images with antibodies against Htr2a (red) and Desmin (green) (**a**–**h**), or Htr2c (red) and Uchl1 (green) (**i**–**p**) along nuclei staining using DAPI (blue). U, urothelia, L, lamina propria, and D, detrusor muscle layers. Bars, 100 µm (**a**, **e**, **i** and **m**) and 25 µm (**d**, **h**, **l **and **p**). Figures were prepared using Adobe Photoshop CS6.

In order to explore the potential molecular candidates contributing to constipation-induced detrusor overactivity, qRT-PCR analyses were performed for the genes involved in neuromuscular function in the bladder, as well as the genes associated with bladder overactivity^26–28,33^. Three genes, 5-HT receptor subtype 2a and 2c (Htr2a and Htr2c) and Transient receptor potential vanilloid 2 (Trpv2) were significantly upregulated in the constipation group in comparison to the control group (Fig. 5A). No significant changes in expression level were observed in other tested genes including muscle contractile factors (Desmin, myosin light chain kinase, Mylk, and myosin heavy chain, Mhc), ion channels (voltage-gated calcium channels, Cav1.2, Cav 3.1 and Cav 3.2, and potassium channels, Bkα, Bkβ1 and Bkβ4), neurotransmitter receptors (muscarinic receptors, Chrm2 and Chrm3, adrenergic receptors, Adrβ2 and Adrβ3, and purinoceptors, P2x1-3,7), and a cholinergic/motor neuron marker (Chat). No changes in the expression level in other classes of 5-HT receptors, Htr1, Htr3, Htr4 and Htr7, and Trp channels, Trpv1, Trpv4,Trpa1 and Trpm8, which are also reported to be expressed in the human and murine bladders, were noted^28,33,34^. Western blotting revealed that Htr2a and Htr2c proteins were elevated by 1.5- and 1.4-fold (p = 0.032 and p = 0.042) at the protein level in the bladder after four days of functional constipation (Fig. 5B,C). We could not determine the level of Trpv2 protein in the bladder as three tested commercially available antibodies did not detect any specific signal with the molecular weight close to the predicted (86 kDa) even for the protein samples from mouse spleen used as a positive control. Immunofluorescence labelling detected Htr2a immunoreactivity (IR) in the urothelial cell cytoplasm and in the detrusor layer in the bladders from the control group (Fig. 6a–d). By contrast, the Htr2a IR was more robust in the constipation group than that in the control group (Fig. 6e–h). The Htr2a IR in the detrusor layer was in a small punctate pattern, appeared to be on the edge of detrusor cells labelled with the antibody against Desmin. The Desmin IR was observed in the detrusor myocytes at the similar level in both groups. The Htr2c IR was distributed throughout the cytoplasm of detrusor myocytes, while no signal in the urothelial and lamina propria layers (Fig. 6i–p). The Htr2c IR did not show overlap with the IR with the antibody against Uchl1 (also known as Pgp9.5), a pan-neuronal marker. The Uchl1 IR was observed mainly in the lamina propria layer and between the muscle bundles in the detrusor layer in similar manner in both groups of mice. The Htr2c IR signal was more intense in the bladders from the constipation group compared with the control group. These results demonstrate that functional constipation induced an upregulation of 5-HT receptors, Htr2a and Htr2c in the urinary bladder.

### 5-HT exacerbated constipation-induced detrusor overactivity

**Figure 7:**
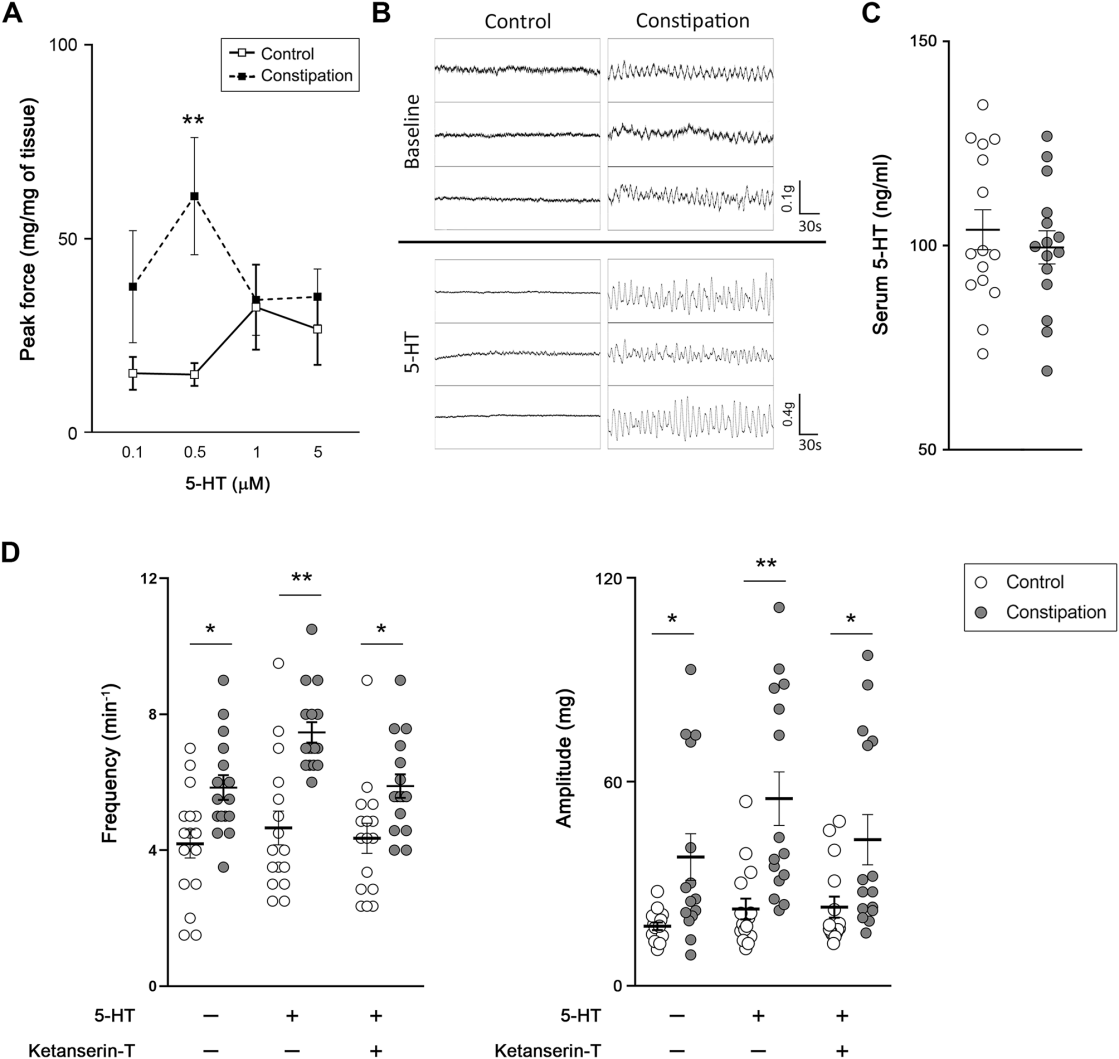
The effect of 5-HT on the bladder strips. (**A**) Peak contractile force in response to 5-HT. (**B**) Representative traces of basal activity of the bladder strips from each group before and after incubation with 0.5 µM 5-HT (upper and lower panels, respectively). (**C**) Serum 5-HT level from each group. the amplitude, and the frequency of spontaneous contractions of the bladder strips. D, the frequency (left) and amplitude (right) of spontaneous contractions of the bladder strips in presence or absence of 5-HT and ketanserin (Htr2 antagonist). Open and grey circles represent the control and the constipation groups (n = 16 per group), respectively. Mean ± SE, *p < 0.05, **p < 0.005 between the groups. Figures were prepared using Adobe Photoshop CS6 and GraphPad Prism 8.4.3 (https://www.graphpad.com/scientific-software/prism/).

To investigate how the Htr2 upregulation in the bladder induced by functional constipation impacts detrusor function, the responses of the bladder strips to 5-HT were evaluated. The 5-HT caused a small contractile response that was less than half of the response to EFS at 2 Hz, with a trend of higher force produced in the constipation group especially at lower concentrations (0.1 and 0.5 µM) compared to the control group (Fig. 7A). On the contrary, the baseline spontaneous activity of the bladder strips was profoundly affected by pre-incubation with 5-HT (Fig. 7B). Serum 5-HT concentration showed no difference between control and the constipation group (104 ± 5 vs. 96 ± 4 ng/ml, equal to 0.59 ± 0.03 vs. 0.57 ± 0.02 µM, p = 0.502) measured by ELISA (Fig. 7C), which correspond to the normal serum 5-HT level (101–283 ng/ml equals to 0.57–1.61 µM)^35^. Accordingly, we selected the concentration of 0.5 µM to further examine the effect of 5-HT on the bladder strips. Pre-incubation of the bladder strips with 0.5 µM 5-HT showed the similar pattern as that without 5-HT, more frequent and larger spontaneous contractions in the constipation group compared to those in control group (4.7 ± 0.5 vs. 7.5 ± 0.3 min^−1^, p < 0.0001, and 22.6 ± 3.0 vs. 55.1 ± 4.6 mg, p < 0.0001, respectively) (Fig. 7D). In comparison to that in absence of 5-HT, the change in the amplitude was statistically significant in both the control (1.3-fold, p = 0.043) and the constipation groups (2.1-fold, p = 0.019). The change in the frequency of spontaneous contractions was statistically significant in the constipation group (1.3-fold, p < 0.0001) but not in the control group (1.2-fold, p = 0.220). The selective Htr2 receptor antagonist, ketanserin (+)-tartrate^31^ treatment in presence of 5-HT reversed the spontaneous contraction responses similar to those in absence of 5-HT in both control and the constipation groups (4.0 ± 0.4 vs. 5.8 ± 0.3 min^−1^, p = 0.003, and 23.1 ± 3.0 vs. 42.9 ± 4.6 mg, p = 0.020). The changes of spontaneous contractions between with and without ketanserin were statistically significant in both the frequency (0.8-fold, p = 0.001) and the amplitude (0.8-fold, p = 0.008) in the constipation groups, but not in the control group (0.9- and 1.1-fold, p > 0.05).

## Discussion

In the present study, we investigated the impact of functional constipation onto the urinary bladder at histological, physiological, and molecular levels. The murine model employed in this study recapitulated clinical and pathological features of functional constipation in children including faecal impaction, faecal incontinence, colorectal distension, and growth retardation^5,36^. Spontaneous voiding behaviour tests demonstrated that constipation led to urinary frequency with decreased volumes per-void in mice. It is possible that large stool in the colorectum takes up abdominal space and prevents the bladder to expand fully, therefore contributing to the decreased functional bladder capacity. Cystometry, an independent in vivo bladder function test, confirmed urinary frequency accompanied with a decreased functional bladder capacity in the constipation group. Additionally, increased NVC suggested bladder overactivity. These results suggested that constipation at young age can induce LUTS and contribute to the development of BBD. During urodynamic tests in awake mice, the control animals usually moved to a corner of the cage immediately before they started voiding as a part of voiding behaviour in mice as described previously^37^. However, the same behaviour was observed only in about half of the micturition cycles recorded in each mouse with constipation. We consider that this behavioural phenotype in mice with constipation was a manifestation of urinary urgency and/or incontinence, another LUTS common in BBD cases.

Constipation is also associated with increased occurrence of UTI in children, and UTI can cause symptoms similar to those of overactive bladder^38^. The urinalysis results showed normal parameters for both groups, indicating that UTI, and bladder lesions were not responsible for LUTS observed in the constipation group. Cystometry showed that the voided volume was approximately equal to the volume infused in each micturition cycle, and no intermittent void was detected in both groups of mice. Altogether, these results suggest that neither bladder outlet obstruction nor detrusor-sphincter dyssynergia developed in the constipated animals tested in urodynamic study. Histological examination also confirmed the absence of detrusor hypertrophy or fibrosis in the bladder, suggesting no bladder outlet obstruction developed along constipation in this study^32^. Based on the current results, we propose three potential explanations for constipation-induced bladder overactivity observed during awake cystometry. First, the bladder experienced an additional external pressure from the overdistended colorectum, which contributed to the rises in intravesical pressure without causing micturition (NVCs). Second, the convergent neurons that receive afferent inputs from both the colon and the bladder were likely continuously stimulated during constipation and sent aberrant signals to the bladder causing an increase in sensation and overactive symptoms. Studies has been shown that colorectal distension or inflammation alters bladder sensations and detrusor activities, and vice versa in both humans and experimental animals^39^, suggesting the existence of "cross-organ sensitization" between the colon and bladder. This might be one of the reasons why successful treatment of constipation leads to an improvement of LUTS in many patients as previously reported^8,19^. However, not all children with BBD improve LUTS after successful resolution of constipation^21,22^. This prompted us to hypothesize the third possibility that constipation, especially in chronic condition, may trigger alterations in the bladder physiology itself (e.g., release of certain neurotransmitters, receptors, ion channels, and other molecules which regulate detrusor function). Our physiological studies in vitro revealed a prominent augmentation of spontaneous contractions of the bladder strips alongside enhanced spontaneous excitation of the detrusor muscle cells in mice with constipation. These results provided evidence that constipation negatively impacted the detrusor itself, and likely altered its physiology independent from the neural inputs. On the other hand, the detrusor contractile responses evoked by EFS, carbachol and KCl as well as the proportion of purinergic and muscarinic contribution to EFS-triggered contractility did not altered following constipation. This result suggested that constipation had no significant impact on the contractile apparatus, the balance between excitatory and inhibitory neurotransmitter release as a whole, or their receptors in the bladder.

Molecular studies revealed comparable expression levels of many genes involved in regulation of detrusor contractile function including muscle contractile apparatus, ion channels, neurotransmitter receptors as well as a cholinergic neuron marker in the bladder, corresponding to the data from physiological evaluation of the bladder. Yet, two 5-HT receptors, Htr2a and Htr2c, were significantly upregulated in the bladder following four days of constipation. Accumulating evidence suggests that 5-HT contributes to LUT function by modulating the activity of the detrusor, external sphincter, urothelium, and neural pathways at both central and peripheral levels^33,34,40^. The effect of selective serotonin uptake inhibitors (SSRIs) as well as agents acts on 5-HT receptors on LUT function has been extensively studied in both clinical and experimental settings, however, demonstrated contradictory results^41–43^. The discrepancies among studies may be due to differences in the pharmacological properties, affinities to different receptors of each agent, availability of receptors, and age and gender of study cohorts^42–44^. Htr2 receptors couple to G_q/11_ protein which activates inositol 1,4,5-triphosphate pathway leading to intracellular Ca^2+^ increase^33^. Our in vitro experiments demonstrated 5-HT at 0.5 µM further augmented overactivity in the bladder strips from the constipation group, which was reversed by the Htr2 antagonist. These results suggest that the increase in the functional Htr2 receptors expressed in the bladder contribute to the constipation-induced detrusor overactivity and related LUTS at peripheral level (in situ). This notion agrees with the association between bladder hyperactivity and Htr2 upregulation in the bladder described previously^44^. Both groups of animals had a normal level of serum 5-HT and the comparable expression of 5-HT transporter, Sert, in the bladder, suggesting that 5-HT bioavailability was unaffected by constipation. Therefore, the detrusor cells could be activated and contract spontaneously at the normal level of 5-HT through Htr2 following a period of constipation. Other 5-HT receptor subtypes (Htr1, Htr3, Htr4 and Htr7) did not show difference in the expression level in the bladder following constipation, suggesting their contribution to 5-HT induced detrusor hyperactivity may be minor^28,33,34^.

The comparable intensity and distribution of Uchl1 IR in the bladders between the two groups indicate that constipation did not alter bladder innervation at appreciable level. The bladder strips from both groups generated equal contractile responses to EFS alongside the equal level of Chat gene expression, suggesting that the proportion of motor and sensory neurons in the bladder was not affected following constipation. Lines of evidence demonstrated that Htr3 plays a predominant role in bladder afferent firing and micturition reflexes at spinal and spinobulbospinal levels^40,45,46^, consequently, an elevated activation of Htr3 pathway facilitates bladder overactivity. Our data showed an equal level of Htr3a receptor expression in the bladder from both groups, suggesting the involvement of Htr3 pathway in bladder overactivity observed in the constipation group to be minor. However, it is possible that constipation induced changes in afferent activity at the level of dorsal root ganglia or spinal cord, and therefore contributes to bladder overactivity observed in the constipation group. We did not address this hypothesis in this study, and it needs to be evaluated in future studies.

An ion channel, Trpv2 also showed a significant elevation in the bladder following four days of constipation. Tprv2 is a member of transient receptor potential (TRP) cation channel family with high Ca^2+^ permeability, and is expressed in the bladder along with several other TRP channels including Trpv1, Trpv4, Trpa1, and Trpm8^28^. TRP channels are expressed in nerve fibres and urothelium in the bladder wall, and suggested to act as sensors of stretch and/or chemical irritation^28^. Yet, the functional significance of Trpv2 in bladder sensation is considered to be minor as the receptor was shown to be not essential for heat or mechanical nociception or hypersensitivity in the adult mouse. On the other hand, several lines of evidence indicate that Trpv2 plays a role in Ca^2+^ influx in different types of myocytes, and there is an association between an upregulation of Trpv2 and abnormal or leaky Ca^2+^ influx in pathologic skeletal muscle^47^. Since Trpv2 is expressed in detrusor cells in addition to nerves and urothelium^28^, we speculated that the upregulation of this receptor may contribute to the elevated spontaneous Ca^2+^ transients in detrusor cells and associated unstable baseline detrusor excitability detected in the constipation group. This conjecture requires to be tested in future studies.

The present study provides initial evidence of association between functional constipation and LUTD, including the changes in detrusor responses to different stimuli and symptoms of bladder overactivity. The elevated expression of Htr2 and Trpv2 in the urinary bladder might account for impaired intracellular Ca^2+^ regulation in the detrusor myocytes, and be associate with the persistence of LUTS even after successful treatments of constipation in BBD^20–22^. The data obtained in this study suggest that local administration of selective antagonists of Htr2 and Trpv2 may offer therapeutic options for BBD patients. Future studies focused on long-term effects of functional constipation on LUT function, as well as a possible prevention and reversibility/progression, will allow for deeper understanding of the mechanisms underlying BBD.

## Supplementary Information

Supplementary Information.
