# Supplementary material for: Functional constipation induces bladder overactivity associated with upregulations of Htr2 and Trpv2 pathways
Source: Sci Rep. 2021 Jan 13;11:1149. doi: 10.1038/s41598-020-80794-0 (PMC7806916; doi:10.1038/s41598-020-80794-0)

Functional constipation induces bladder overactivity associated with upregulations of Htr2 and Trpv2 pathways

Nao Iguchi[1], Alonso Carrasco Jr.[2,3], Alison X Xie[1], Ricardo H Pineda[1], Anna P Malykhina[1] and Duncan T Wilcox[1,2,*]

[1]Division of Urology, Department of Surgery, University of Colorado Denver School of Medicine, 12700 E 19th Avenue, Aurora, CO 80045; USA, [2]Children's Hospital Colorado, 13123 E 16th Avenue, Aurora, CO 80045, USA, [3]Children's Mercy Kansas City, 2401 Gillham Rd, Kansas City, MO 64108, USA

Number of tables: 3

Number of figures: 7

Number of supplementary figures: 2

Grant Support: This study was supported by University of Colorado, School of Medicine Academic Enrichment Seed Funds (D.T.W.) and Ponzio Family Endowment Fund (D.T.W.).

*Correspondence: Children's Hospital Colorado, 13123 E 16th Avenue, B463, Aurora, Colorado 80045, USA (Telephone: +1-720-777-3926, E-mail: Duncan.Wilcox@childrenscolorado.org).

Supplementary data 1

Validation of specificity for anti-Htr2c antibody [LS-C386171].

Expression level in the NCBI database
- Cerebellum, Pons +++
- Testis +
- Bladder, Lung ±
- Intestine -

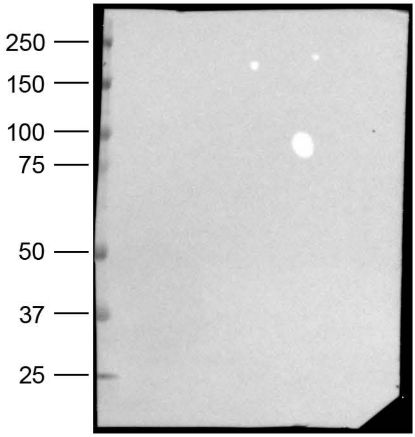

Bright field full blot image

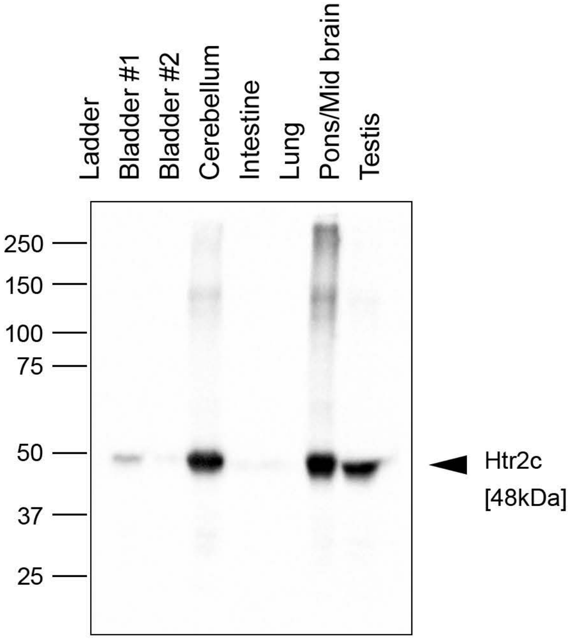

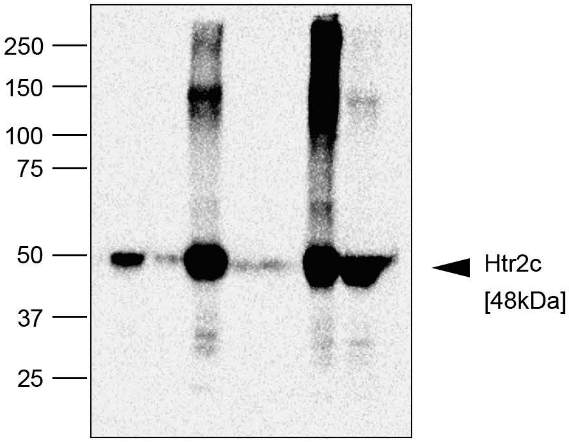

Long exposure image

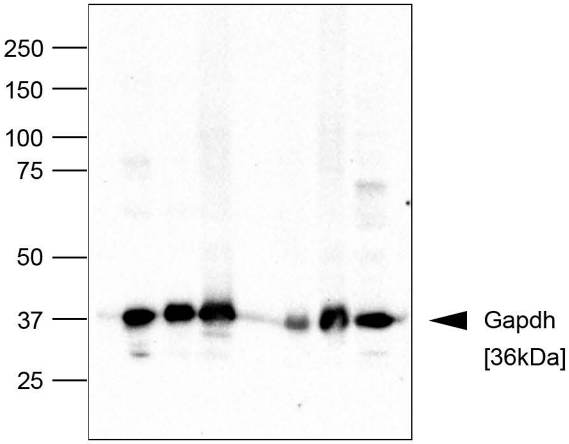

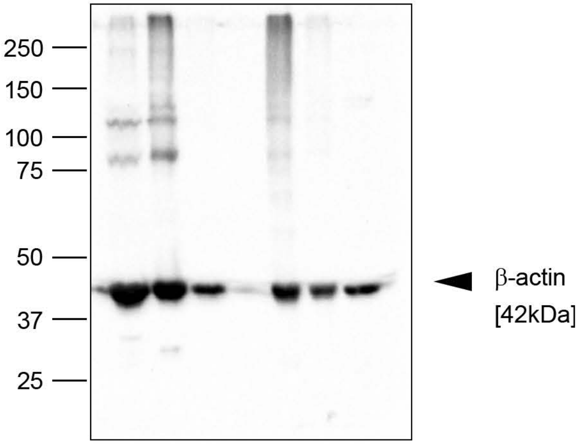

Supplementary data 2. Full size Western blot images with the bright field image of the filters.

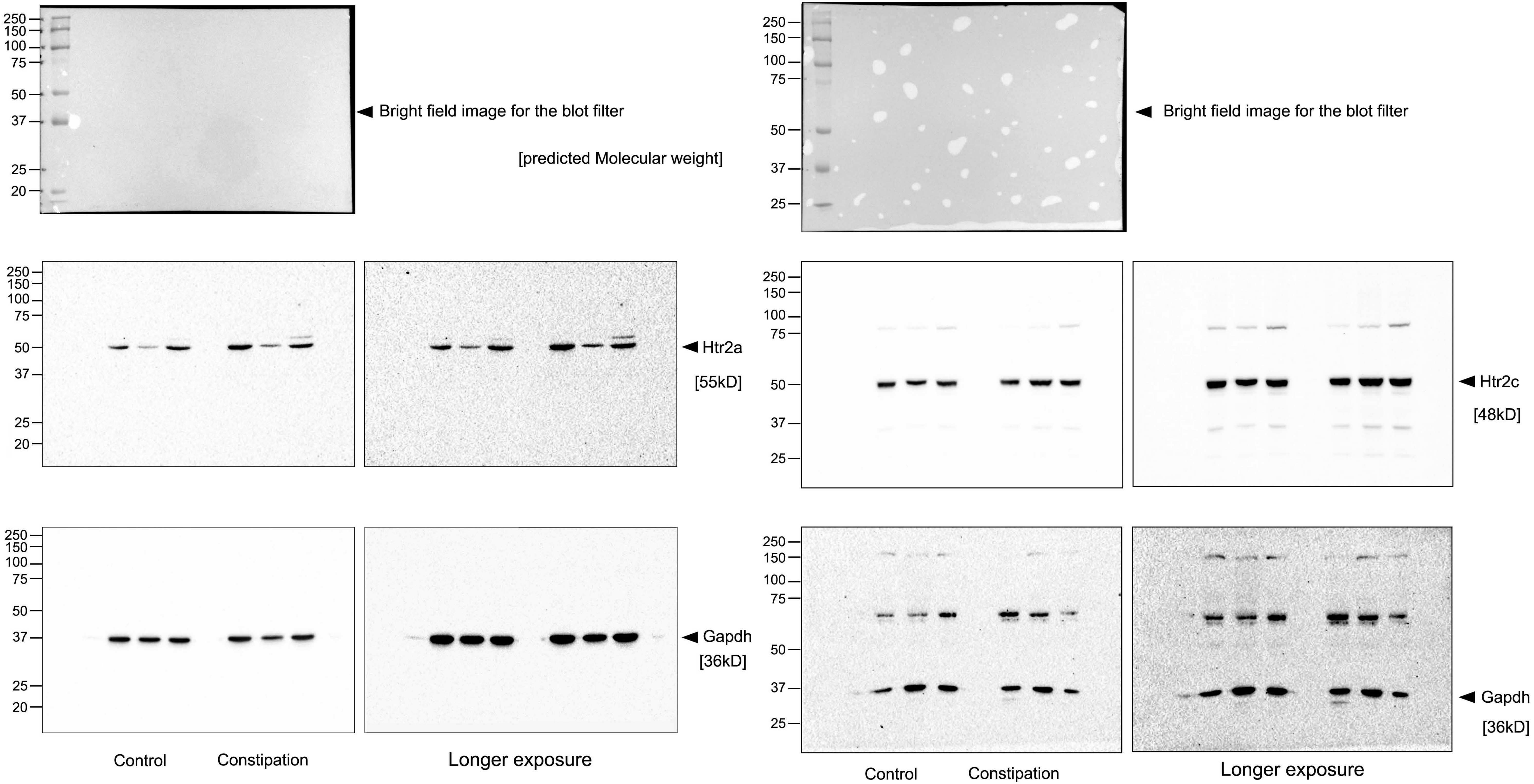

Supplement: Supplementary file 1 — Supplementary Information. [file 41598_2020_80794_MOESM1_ESM.pdf]
